# Supplementary figures and images for: Comparison of FORCE trained spiking and rate neural networks shows spiking networks learn slowly with noisy, cross-trial firing rates
Source: PLoS Comput Biol. 2025 Jul 21;21(7):e1013224. doi: 10.1371/journal.pcbi.1013224 (PMC12367184; doi:10.1371/journal.pcbi.1013224)

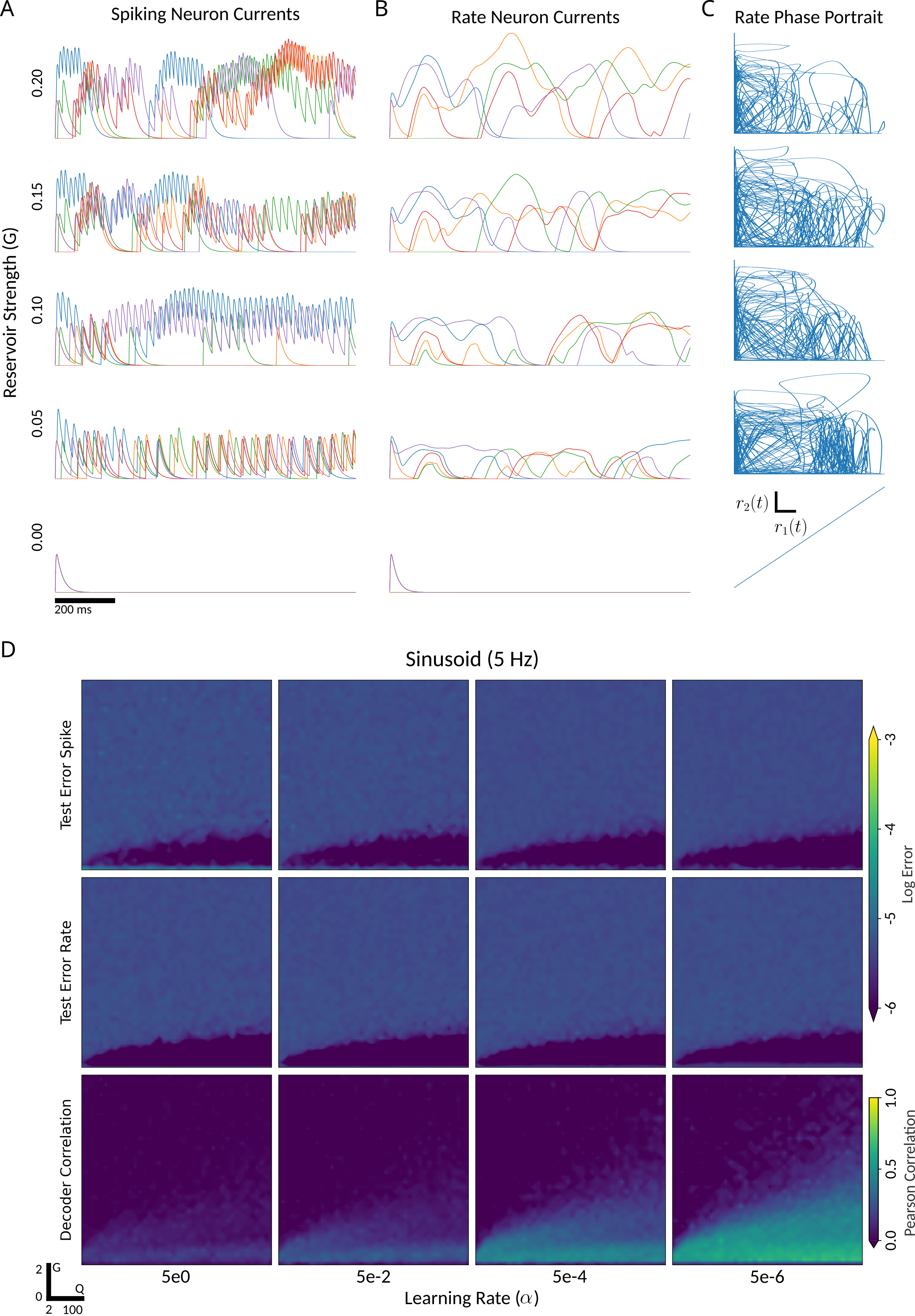

Supplement: S1 Fig — A-B Neural currents for networks of 200 LIF neurons and their corresponding LIF-matched rate neurons, demonstrated chaotic behaviour for reservoir strength parameter G>0. C Phase portrait of the first two neurons in the rate network simulated for 50 s, displaying chaotic behaviour for G>0. D Networks of 2000 LIF and LIF-matched rate neurons were trained over a 40×40(Q,G) parameter grid with G∈[0,2] and Q∈[2,100]. For sufficiently large G, neither network was able to learn. (TIFF) [file pcbi.1013224.s001.tiff]

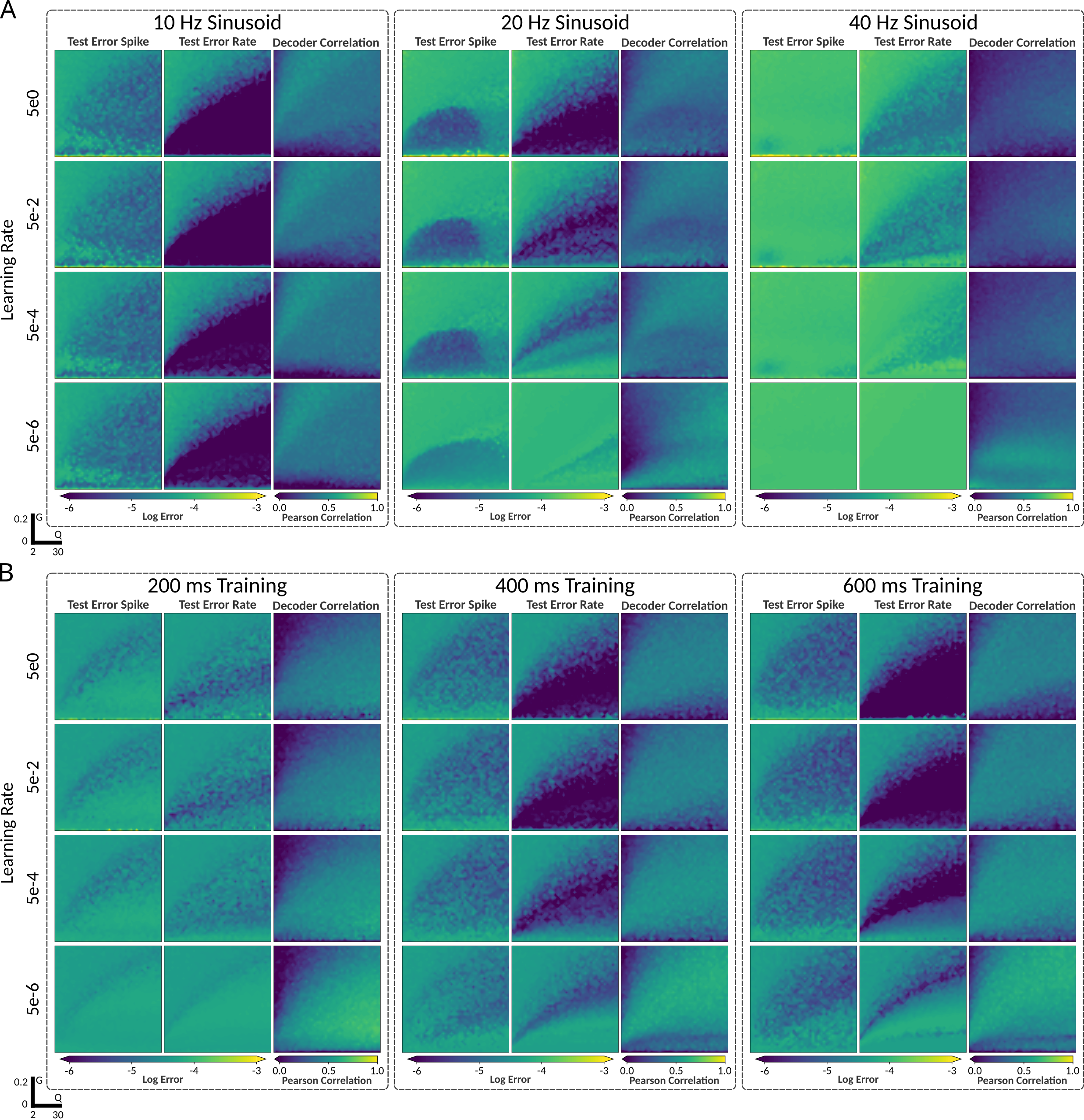

Supplement: S2 Fig — A Networks of 2000 LIF and LIF-matched rate neurons were trained to generate sine waves of increasing frequency. As the frequency increased, both networks exhibited reduced ability to learn the supervisor. B Networks of 2000 LIF and LIF-matched rate neurons were trained to generate a 5 Hz sine wave with varying training durations. When the training period was very short (containing only a single cycle of the supervisor), neither network was able to learn. (TIFF) [file pcbi.1013224.s002.tiff]

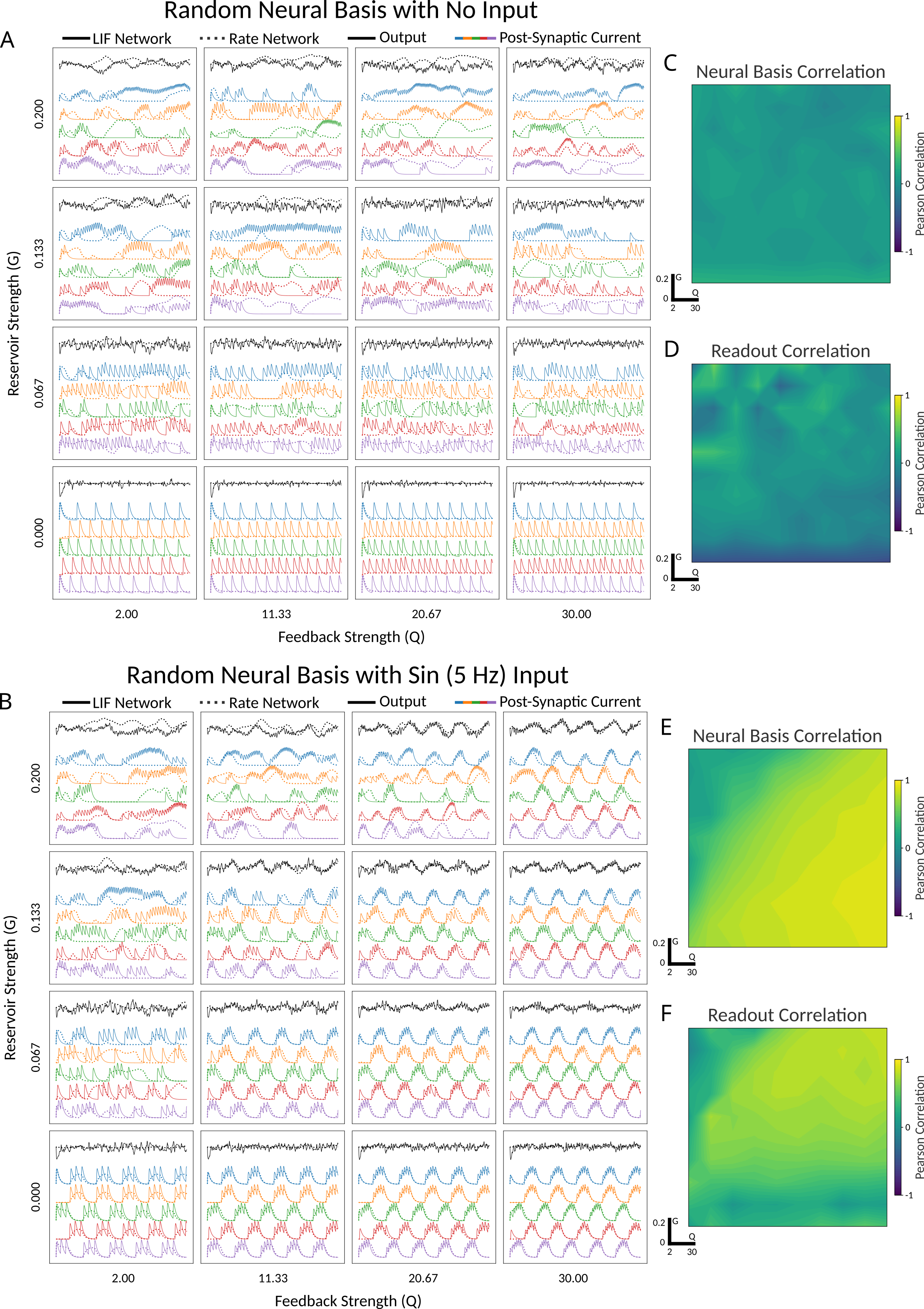

Supplement: S3 Fig — Networks of 2000 LIF and LIF-matched rate neurons were simulated for 1s across different reservoir strengths (G) and feedback strengths (Q), both with and without driving input. In the absence of input, both networks exhibited chaotic dynamics and low cross-network correlations. When driven, the cross-network readout and neural bases became correlated. A–B Sample readouts and neural basis elements from both networks. C–F Cross-network correlations of sampled neural bases and readouts. (TIFF) [file pcbi.1013224.s003.tiff]

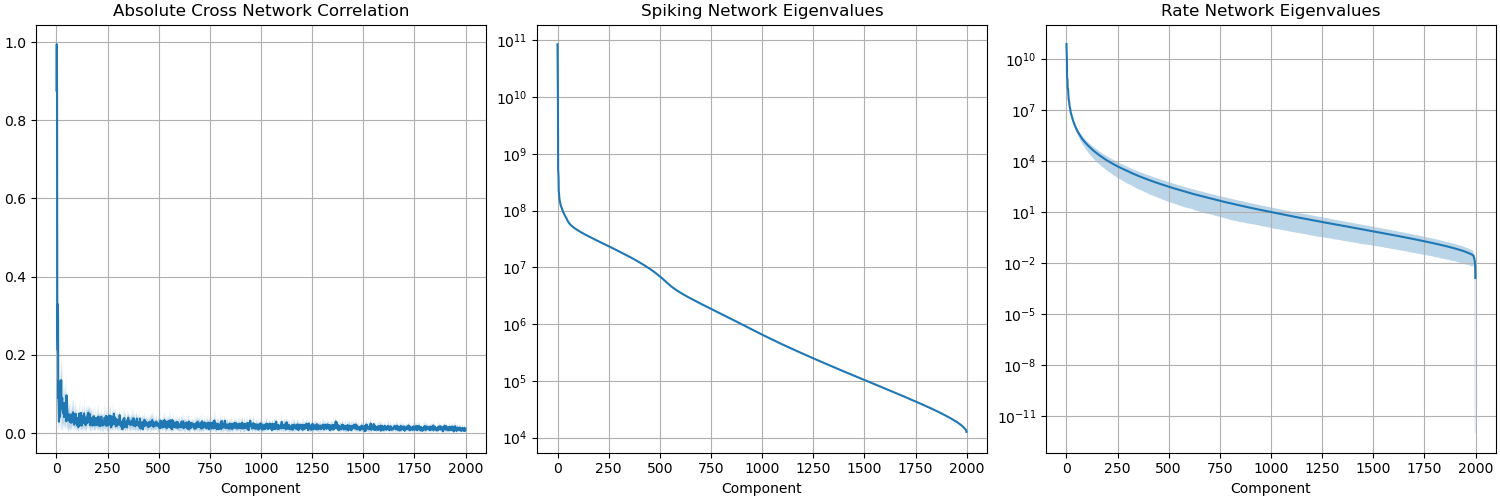

Supplement: S4 Fig — Networks of 2000 LIF and LIF-matched rate networks were trained with FORCE on the 5 Hz sinusoidal supervisor for 4s with learning rate α=5e-6, reservoir strengths G = 0.1, and feedback strengths Q = 15, for 10 different seed values. We then plot the averaged: absolute correlation in the orthogonal basis elements, LIF eigenvalue, and Rate eigenvalue. The shaded regions represent the standard deviation. The lower order orthogonal basis elements have much higher associated eigenvalues and so explain much more of the variability in the original basis. The early basis elements are also highly correlated across networks types. (TIFF) [file pcbi.1013224.s004.tiff]
