# Supplementary material for: Comparison of FORCE trained spiking and rate neural networks shows spiking networks learn slowly with noisy, cross-trial firing rates
Source: PLoS Comput Biol. 2025 Jul 21;21(7):e1013224. doi: 10.1371/journal.pcbi.1013224 (PMC12367184; doi:10.1371/journal.pcbi.1013224)
Supplement: S1 Text — Methods for S3 Fig. (PDF) [file pcbi.1013224.s005.pdf]

S3 Fig Methods

Each network used the same randomly initialized decoder, $\boldsymbol{\phi}^{rand}$, sampled from a normal distribution $\mathcal{N}\left(0, N^{-1}\right)$ with zero mean and standard deviation N^{-1}. To ensure an exactly zero mean, the sample mean was subtracted from the weights. This was done by first sampling $\tilde{\boldsymbol{\phi}}_{rand} \sim \mathcal{N}\left(0, N^{-1}\right)$ and then defining:

$$\phi_i^{rand} = \tilde{\phi}_i^{rand} - \frac{1}{N}\sum_{j=1}^{N} \tilde{\phi}_j^{rand}. \tag{1}$$

The driving input $y(t)$ was introduced through an input weight vector $\boldsymbol{\psi} \in \mathbb{R}^N$, scaled by the feedback strength parameter Q. The input current the ith neuron was thus defined by:

$$I_i^{S/R}(t) = \sum_{j=1}^{N} \omega_{ij}^{S/R} r_j^{S/R}(t) + I_{bias} + Q\psi_i y(t). \tag{2}$$

Each network used the same $\boldsymbol{\psi}$, sampled from a uniform distribution over the interval $[-1, 1]$. The feedback and reservoir weights were generated as described in the Network Models section of the results.

The neural basis correlation was approximated by taking a random sample of 20 neurons, computing the cross-network Pearson correlation, and then taking the average across the 20 samples:

$$\rho\left(\boldsymbol{r}^S, \boldsymbol{r}^R\right) = \frac{1}{20}\sum_{i=1}^{20} \rho(\boldsymbol{r}_i^S, \boldsymbol{r}_i^R), \tag{3}$$

where ρ is the Pearson correlation. The matrices $\boldsymbol{r}^S$ and $\boldsymbol{r}^R$, are the time-sampled neural bases from the LIF and LIF-matched rate networks respectively. The readout correlation was computed by the Pearson correlation between the time-sampled time-series of decoded outputs, $\hat{x}^S(t)$ and $\hat{x}^R(t)$, from the two networks.
