# Supplementary material for: Comparison of FORCE trained spiking and rate neural networks shows spiking networks learn slowly with noisy, cross-trial firing rates
Source: PLoS Comput Biol. 2025 Jul 21;21(7):e1013224. doi: 10.1371/journal.pcbi.1013224 (PMC12367184; doi:10.1371/journal.pcbi.1013224)
Supplement: Attachment — Submitted filename: PlosCompBio Review.pdf [file pcbi.1013224.s006.pdf]

Dear Authors,

First, I enjoyed reading and engaging with the question central to your work. Your attempt to highlight how LIF networks trained by FORCE end up in a rate-encoding regime and have difficulty dealing with high learning rate (in comparison to LIF-Rate neurons) is valuable for the research community and it is clear that you have put in a lot of work to explain the observations that you see.

I do have a number of questions and suggestions that I shall structure below, from largest to smallest in scale and with headings to help any discussion. Most importantly, I am concerned that your `equivalent' networks are not truly equivalent in certain regimes and that you may wish to put much more emphasis on this.

<u>Major Concern: The reality of equivalence between your rate and spiking LIF neurons</u>

My biggest concern with this work relates to how certain one should be of the equivalence between your rate and spiking LIF neurons. Your paper starts by showing that, when trained slowly, these `equivalent' networks perform … well equivalently. But thereafter goes on to show that this is not the case. I have a number of things I'd be curious to know:

1) The equivalent rate formulation is only true for a fixed input (as far as I know). This should be mentioned as a possible source of error I believe. Happy to be proven wrong on this front.
2) If you randomly initialize a set of networks (spiking and rate-LIF), what is the correlation in the readout signal between these? This would help to give context for the earlier figures (3 and 4) about how similar (or equivalent) these networks really are on average. Currently I am concerned that they are actually quite dissimilar in practice.
3) Figure 5 seems to show that there are locations in parameter (weight) space, where the equivalence between the spiking and rate networks completely breaks down. In fact, it even suggests a directional relation, that the rate networks can well approximate spiking network behaviour but not the other way around. Is this true? Can one determine or say anything (statistically or in terms of weight magnitudes even) about where the rate networks stop behaving like the spiking networks? This would be a core crux for your argument.
4) On the whole, it seems that your conclusion is that FORCE learning in rate-LIF networks is encouraged to find a different set of solutions due to the noise structure in these networks being quite different to those of spiking networks. Might you be able to give some additional contribution to the field here by suggesting how to improve the alignment between the rate and spiking-LIF models? Here it seems that the rate-LIF model is estimating only a mean firing rate and not a variance, though I am aware that theoretical developments can be challenging to pursue.

Should this work answer a few more of these core concerns, I would be happy to upgrade my recommendation. Below are some more minor suggestions (though they are important for quality).

<u>Minor points of attention:</u>

1) Tau_{ref} is given in Table 1, but never referred to in the text. I believe it should be the refractory period? (perhaps there is a confusion due to the existence of both tau_r and tau_{ref})

2) The equations (1-5 and onwards) are a little confusing with respect to units - also true for Table 1. Inputs are presented as currents in Table 1 but do not have the right unit (should be volts rather than amps). Can be easily corrected by including a resistance or conductance in the equations - or equivalently by redefining your currents as input voltages.
3) Page 7 of the main text refers to Figure 6 when I believe it intends to refer to Figure 7.
4) On page 11 (Materials and Methods), the spiking reset mechanism is indicated by a right arrow from v_i to v_{reset}, as far as I'm aware this should be a left-arrow indicating assignment rather than tending.

Best of luck with the next steps.
