# Supplementary material for: Comparison of FORCE trained spiking and rate neural networks shows spiking networks learn slowly with noisy, cross-trial firing rates
Source: PLoS Comput Biol. 2025 Jul 21;21(7):e1013224. doi: 10.1371/journal.pcbi.1013224 (PMC12367184; doi:10.1371/journal.pcbi.1013224)
Supplement: Attachment — Submitted filename: response_referees.pdf [file pcbi.1013224.s008.pdf]

We thank all three referees for their thorough and insightful reviews. We have implemented many of the requested changes, including new simulations, new supplementary figures, and rewriting many parts of the manuscript for clarity. We outline our responses below, and to facilitate the revisions process, we have provided a latex-diffed PDF which highlights the changes made in the manuscript. We look forward to hearing their responses.

Reviewer #1:
The study compares two types of networks trained under FORCE learning: a spiking LIF network and a rate-based version of the former. The rate-based approximation relies on a classical derivation: with constant current, the inter spike interval (ISI) of deterministic LIF is constant. Both networks are trained on the same toy tasks: Lorenz attractor, bit-flip or reproducing period inputs. The two networks are matching very strongly in the specific regime considered. The most prominent difference highlighted is that rate-based networks learn better under high learning rates.

The match between spiking and non-spiking networks here is somewhat remarkable given that it is uneasy to find an accurate one-to-one mapping between spiking networks are rates. However the derivation relies on the classical derivation of stationary ISI for the case of a single neuron and this interesting extension to the network dynamics is easily explainable since the current varies slowly. I believe this is engineered in the simulations of this paper and would not be true for most spiking models (see point A). Also the scope of spiking networks studying here is restricted and unlikely to other more useful applications of spiking network models (see point B).

Major Points

1. The classical results on stationary spike-intervals in deterministic LIF networks is sufficient here by design for two reasons: (1) the target signals are relatively slow to match the quasi-stationary current and ISI hypothesis, (2) the network are set in a very sub-chaotic state (G~0.1) probably near the mean-field limit where weights are weak and firing is driven by collective activity and reducing the transient effects of isolated spikes (see [1] for a theoretical mapping of stochastic spiking networks and rate based networks in the mean field regime). The point (2) is probably even consolidated by FORCE learning which projects back a low-dimensional input using low-dimensional factors.

So one essential problem here is that (2) is not commented on or highlighted, but I strongly suspect this is an important assumption here. An easy check would be to study higher ranges for G which are closer to the chaotic regime (G~1 is the parametrization is nice, otherwise

showing that dynamics start to vary in a chaotic fashion at some point). The whole point of the paper is strongly defeated if the distances between spiking and rate based networks decreases although both networks solve the tasks with near-chaotic G which is the most standard choice.

We thank the referee for mentioning the transition to chaos, and the slowness of the signals. The referee is correct that we should show that the networks trained are indeed chaotic in the parameter regime. While G is small, the parameterization is "not nice" as the referee mentions and both the spiking and the rate reservoir are chaotic without any supervisor. To address the referee's criticism we have:

- Conducted simulations in the g range that we consider (G~0.1 as the referee mentions) and show that the networks are indeed chaotic through direct simulation, We remark that, as the referee states, the G~1 chaos transition only occurs when the parameterization is nice (the system is rescaled), and this is not the case here. **We've verified the chaotic state for G~0.1 and smaller (in this parameterization) which is New Supplementary Figure S1.**
- Further, we've shown that neither the rate network nor the spiking network learn for higher values of g. If the chaotic dynamics of the reservoir are too strong, it is difficult for a supervisor to stabilize the system in all cases. **We've added these results both in text and as New Supplementary Figure S1**
- Additionally, we now also look at faster supervisors and compare both the rate and spiking networks. **This is shown in the new Supplementary Figure S2 with faster sinusoidal oscillators.**

2. I find the scope of analysis quite limited in the paper. Only the case of FORCE learning is studied under tasks which are relatively very simple in comparison with contemporary application of spiking networks. For instance the Od to the Joy task requires repeating (hence "overfitting") a periodic signal and does not require generalization. This optimization is far more trivial in comparison with the fitting to neural recordings [2] or machine learning applications as done with surrogate gradient techniques). Typically it is likely that the quasi-stationary current assumption would not hold in these harder cases.

Yes, this paper is primarily analyzing FORCE trained spiking/rate matched reservoirs to analyze how these two types of networks perform with the exact same learning regime/parameters. We remark that while the tasks may appear simple, the network is in fact learning multiple different types of dynamical systems, such as oscillators (sinusoidal tasks), multi-stable states (the pitchfork normal form), and low-dimensional chaotic dynamics (Lorenz system) which are

standard benchmark tasks. We also remark that the chaotic time series tasks are in fact challenging and the standard benchmark for various applications of reservoir computing.

We greatly appreciate the reviewer's suggestion to explore more sophisticated optimization methods, particularly surrogate gradient descent. We wholeheartedly agree that this is an important direction for future work. We suspect differences would arise between the network types for other learning techniques such as the surrogate gradient methods. However, we believe this would be best addressed in a separate manuscript. The primary focus of this study was to establish the viability of comparing spiking and rate networks as we did and to analyze how the FORCE method trains spiking networks—which revealed that they approximate noisy firing rates. **We now expand on this potential avenue for future work in the discussion section.**

3. The title suggests that the strongest result is that spiking networks do not benefit from fast learning rate. I think this is however mildly surprising, and if this is such a strong result then it could deserve more analysis. I suggest two possible hypotheses: the very fast learning rates in Figure 8 are sufficient to "learn" with hundreds of milliseconds. I wonder whether this alone might not induce fast dynamic changes which are interfering with the quasi-stationary ISI assumptions. Second hypothesis, the spiking network behaves as a "noisy" rate base network so more repetition of the periods are needed to average learning signals.

We appreciate the referees suggestions, to test the first hypothesis, we stopped the learning procedure in both the spiking/rate networks early when the learning rate was fast for the oscillator task. **This is shown in New Supplementary Figure S2.** We agree that the spiking network behaves as a noisy rate network. We would suggest that the slower learning rate effectively increases the "averaging window" over which decoder updates are computed, which stabilizes the updates and counteracts the noisy basis. What's important is that the averaging needs to occur for each update, which is controlled by the learning rate, so simply increasing the training time does not improve stability and learning. **We now discuss this further in the manuscript.**

Reviewer 2:

Dear Authors,

First, I enjoyed reading and engaging with the question central to your work. Your attempt to highlight how LIF networks trained by FORCE end up in a rate-encoding regime and have difficulty dealing with high learning rate (in comparison to LIF-Rate neurons) is valuable for the research community and it is clear that you have put in a lot of work to explain the observations that you see. I do have a number of questions and suggestions that I shall structure below, from largest to smallest in scale and with headings to help any discussion. Most importantly, I am concerned that your `equivalent' networks are not truly equivalent in certain regimes and that you may wish to put much more emphasis on this.

Major Concern: The reality of equivalence between your rate and spiking LIF neurons
My biggest concern with this work relates to how certain one should be of the equivalence between your rate and spiking LIF neurons. Your paper starts by showing that, when trained slowly, these `equivalent' networks perform … well equivalently. But thereafter goes on to show that this is not the case. I have a number of things I'd be curious to know:

Major Points

1) The equivalent rate formulation is only true for a fixed input (as far as I know). This should be mentioned as a possible source of error I believe. Happy to be proven wrong on this front.

We thank the referee for their comment. First, while the correspondence between rates/filtered spikes are true for a fixed input, the firing rate approximation (interchanging rates with filtered spikes) is more generally true for slowly varying currents through dynamical systems averaging, or when the network filters (e.g. synaptic filters) are sufficiently long, which allows the individual spikes to accrue in the post-synaptic current. Indeed, the synaptic filtering functions can be interpreted as a kind of "firing rate" as each spike gets filtered and added to the sum of filters to form a postsynaptic current, which acts like a kernel density estimator for the spike density in time (or the firing rate). **Further, we've now compared the firing rates with a time-varying input in the New Supplementary Figure S3.**

2) If you randomly initialize a set of networks (spiking and rate-LIF), what is the correlation in the readout signal between these? This would help to give context for the earlier figures (3 and 4) about how similar (or equivalent) these networks really are on average. Currently I am concerned that they are actually quite dissimilar in practice.

This is a good suggestion by the referee. This depends on if the reservoir is trained or not. If the reservoir is un-trained, and the readouts are also randomly generated, then as the initial reservoirs (both spiking and rate) are chaotic, even if the spiking network and the rate network are initialized in the same parameter regime, the correlation between the firing rates of these two reservoirs will be low. This is due to slight differences in the rate versus spiking reservoir dynamics that amplify due to the chaotic nature of the reservoir. If, however, the reservoir's (spiking and rate) are both trained on the same task, or both receive the same inputs which can stabilize the chaos, then the correlation between the readout signals can be quite high for non-chaotic supervisors. For chaotic supervisors, eventually the spiking and rate reservoirs will diverge from each other, while still displaying the same chaotic dynamics and attractors. **This is now shown in new Supplementary Figure S3.**

3) Figure 5 seems to show that there are locations in parameter (weight) space, where the equivalence between the spiking and rate networks completely breaks down. In fact, it even suggests a directional relation, that the rate networks can well approximate spiking network behaviour but not the other way around. Is this true? Can one determine or say anything (statistically or in terms of weight magnitudes even) about where the rate networks stop behaving like the spiking networks? This would be a core crux for your argument.

While it may seem like there are areas in the parameter space where equivalence breaks down, there's an important caveat: this only occurs when the learning rate is too fast. When the learning rates are slower, the networks function equivalently (see Figure 4) including in the hyperparameter space of G, Q. This implies that over large areas of parameter space, there are weight solutions that are exchangable between the two networks. However, when the learning rate is faster, the weight (decoder) correlations break down, and the spiking decoder no longer converges to a solution (Figure 8). **For the faster learning rates we've considered, the rate network does appear to have consistently smaller weight magnitudes (as measured by the decoder), which we now mention in the discussion.** We leave an analytical derivation of this point, which relates the weight magnitude to the stability of the spiking versus the rate network as a future point of work.

4) On the whole, it seems that your conclusion is that FORCE learning in rate-LIF networks is encouraged to find a different set of solutions due to the noise structure in these networks being quite different to those of spiking networks. Might you be able to give some additional contribution to the field here by suggesting how to improve the alignment between the rate and spiking-LIF models? Here it seems that the rate-LIF model is estimating only a mean firing rate and not a variance, though I am aware that theoretical developments can be challenging to pursue.

We thank the referee for this suggestion. One key contribution is to recommend slower learning rates when using FORCE training, which results in the rate/spiking networks aligning. We now mention this more explicitly in the discussion for readers to be aware

Yes, we fully agree with this interpretation. The firing rate serves as a modeling approximation of the spiking basis, assuming that the mean number of spikes fired provides a sufficient description of the network while omitting cross-trial variability. Our finding that the learned spiking decoder generally functions in the rate network suggests that the information FORCE uses to train the decoders is largely captured by the firing rate description.

Importantly, our goal is not to construct perfectly equivalent spiking and rate networks but rather to understand the underlying mechanisms by which FORCE trains networks. The development of equivalent networks is simply a natural byproduct of this investigation.

Additionally, we note that the firing rate description used here is averaged over time. Given that we observed some cross-trial correlations in spike times, a more precise description might be a cross-trial averaged firing rate. We hypothesize that the information captured by FORCE represents a blend of these two firing rate descriptions, as the learning matrix P is a running approximation (or average) of the inverse cross-correlation in the neural basis.

Should this work answer a few more of these core concerns, I would be happy to upgrade my recommendation. Below are some more minor suggestions (though they are important for quality).

Minor points:

1) Tau_{ref} is given in Table 1, but never referred to in the text. I believe it should be the refractory period? (perhaps there is a confusion due to the existence of both tau_r and tau_{ref})

Yes, this is the refractory period. **We now refer to it in the text and methods.**

2) The equations (1-5 and onwards) are a little confusing with respect to units - also true for Table 1. Inputs are presented as currents in Table 1 but do not have the right unit (should be volts rather than amps). Can be easily corrected by including a resistance or conductance in the equations - or equivalently by redefining your currents as input voltages.

We have now made the units consistent. We thank the referee for mentioning this.

3) Page 7 of the main text refers to Figure 6 when I believe it intends to refer to Figure 7.

This is now been corrected.

4) On page 11 (Materials and Methods), the spiking reset mechanism is indicated by a right arrow from v_i to v_{reset}, as far as I'm aware this should be a left-arrow indicating assignment rather than tending.

This has now been changed.

Best of luck with the next steps

Thank you!

Reviewer #3:
Overall, I value the paper and I believe it addresses a relevant question, that many of us that have worked (or tried to) with spiking FORCE networks will recognise. I appreciate the methods explanation per figure, which makes it easy for the reader to reproduce the results.

I have two main issues:
Some of the structure is not quite clear. Parts of the system are explained in the results, with a more extensive explanation in the Methods. However, no references are made between the two, which makes it difficult for the reader to understand the 'Network Models' part of the results. (A) For instance, the difference between tau_r and tau_ref is not explained, it is not explained that the spikes are in equation 3 (basically, how to go from v to r), and (B) what is tau_d? (B) For eta and phi the dimensions are given, but it is not explained what they are, and are the brackets in equation 6 a function (i.e. eta is a function of phi) or are they just there for the transpose? (C) What is P (the update role is given, but that it is related to the rate correlation matrix is stated nowhere). In the end, most of this can be deduced from the methods, but it would be nice if it were either explained more explicitly, or contained references to where it is explained. (D) In particular, the learning rate alpha is not in any of the equations, which is very confusing, as this is one of the key points of the paper. (E) The tasks (pitchfork, ode to joy, etc) are also not explained in the results, which makes this hard to follow.

We thank the referee for his comment here as it greatly helps with the readability of the manuscript. To that end, we have now:

A) Explicitly defined what the variables tau_ref, tau_r, tau_d are in the main results
B) Defined what the dimensions of the encoders/decoders are in the main results and explained the decoder notation
C) Defined P in the main results, and its role in the Recursive Least Squares (RLS) updates.
D) Explicitly talked about the learning rates in Recursive Least Squares
E) We have also now explained the different dynamical tasks in the results as well.

A more fundamental issue is that of task dependence. Figure 6 shows clear task dependence: increasing the learning rate alpha (so going from bottom to top) increases the area of convergence, but not for Ode to Joy. So it is not task independent. Yet, in figure 6, 7 and 8 you only go on with the 5 Hz oscillations (which is quite slow for a neural system).

To address the referees response, we have added a new supplementary figure with additional faster supervisors while varying the FORCE learning rate (New Supplementary Figure S2).

The scaling with network size is very interesting, and I can understand that you cannot test this for all G,Q values and tasks, but why was this particular parameter set and task chosen?

The task chosen (the oscillator task) was so that the error metric (root-mean-squared error) could be easily computed. For example, the RMSE can't really be computed in a chaotic supervisor, since the network diverges away from the supervisor even with small differences in their initial condition, and small errors in learning the dynamics. The G/Q parameters were arbitrarily chosen to be inside the region of convergence for FORCE for the sinusoidal supervisor. We have now added this justification to the manuscript.

Do you expect your results to change with different parameter values or with a different task, as we say that the area of convergence is task dependent.

This is an interesting question that the referee poses. We primarily expect that our results may change with the learning algorithm used. FORCE maybe more or less stable to the noise produced by spikes, and therefore requires slower learning rates, while other methods (e.g. surrogate gradient descent) may be more or less amenable to the "noise" behind spike times. **We now mention this explicitly in the discussion.** As for task dependence, most of the tasks we have considered require some reproducibility (consistency) of the spiking basis as they constitute dynamical systems where the network responds consistently to its local position in

state-space. It is possible that if the network were to be trained on time series which can intrinsically use the noise in spikes, the results may differ.

Moreover, next to different dynamical systems, Nicola and Clopath show that their system can also function for different tasks, for instance as a classifier. Maybe the authors could discuss whether their results would also hold for classifier tasks?

We expect similar results to hold for classification-based tasks, as effective classification requires input currents to drive network activity, much like the tasks examined here. In the work by Nicola and Clopath, a high degree of variability was observed in neural voltages for the same inputs—both near and far from the decision boundary. This finding aligns with our observations in this study. **This is now explicitly mentioned in the discussion.**

Finally, trial to trial variability is not always the same as jitter, as in the predictive coding networks of Machens and Denève that the authors cite demonstrate: they have a large trial to trial variability without a loss of information, whereas jitter would increase the error a lot. So I suspect that the results might not always hold over tasks, and that spiking network might perform better for tasks where faster timescales are required. As I understand that it is not feasible to simulate too many tasks and parameters, this should at least be discussed.

We have now discussed the difference between trial to trial variability versus spike jitter explicitly, and have highlighted the differences between the two types of variability (variability in the basis versus variability in the response**.**

We have changed the reference to "spike time jitter" in regards to the work by Machens and Denève to "cross-trial variations in spike time". **Additionally we have elaborated on our discussion of cross-trial variance and its relation to the predictive coding networks.**

Minor points:

I think the term 'LIF-rate' network is confusing: the F in LIF stands for fire, and that is exactly what rate neurons do not do. Could you maybe use a term like 'LIF-matched rate network' or something?

We have now referred to the LIF-Rate network as just the rate network for clarity.

In the introduction, there are some local learning rules in predictive coding networks that I believe should be mentioned, as it shows that the representations can be learned by local Hebbian rules:

Bourdoukan, R., Barrett, D. G. T., Machens, C. K., & Denève, S. (2012). Learning optimal spike-based representations. In P. Bartlett, F. C. N. Pereira, C. J. C. Burges, L. Bottou, & K. Q. Weinberger (Eds.), Advances in Neural Information Processing Systems 25 (pp. 2294–2302).

Brendel, W., Bourdoukan, R., Vertechi, P., Machens, C. K., & Denève, S. (2020). Learning to represent signals spike by spike. PLoS Computational Biology, 16(3), Article 3. https://doi.org/10.1371/journal.pcbi.1007692

We thank the referee for bringing these references to our attention, **which we now add to the main manuscript.**

At the end of the results section, it is not completely clear what you mean here by a basis. Could that be specified more explicitly?

We have now explicitly defined the basis. It's the set of temporal features created by a specific supervisor as its fed through the reservoir during training. In our case specifically, the neural basis for each network is the set of filtered postsynaptic currents.

Figure 4: the fact that the stars are at the edge of your figures, suggests that if you would have chosen a larger range of G and Q values, there might have been even more correlated values. Does the correlation go back up again, or does it keep decreasing? Why was this range of G and Q chosen?

We have now added simulations with larger G/Q values (Supplementary Figure S1). Under these circumstances, neither the rate nor the LIF networks actually converge under larger G/Q regimes.

Fig 4 and 5: why were these alpha values chosen, what was it based on? And from the methods I read that a HDTS signal was used to stabilise the network. This is not trivial to me: could this be explained in the results or discussion? What was is based on, why does that work, why is it needed?

The HDTS signal was originally used in Nicola and Clopath 2017 as additional elements of a supervisor that act as a clock for encoding extremely long signals. **This has now been mentioned explicitly in the discussion.**

In figure 7 there is an orange line that is not mentioned in the caption, whereas there is mention of a green line that is not in the figure

This has now been corrected.

Figure 8a needs a legend (what are the colours?).

A legend has now been added.

I add the pdf with some highlighted typos (as there are no line numbers), and I few sentences I could not follow.

The PDF was greatly appreciated, and we have made the suggested edits by the referee. To facilitate an easier re-review, we have attached a Diff version of the PDF that highlights all changes made by us in the revisions and included line numbers.
