# Supplementary material for: Comparison of FORCE trained spiking and rate neural networks shows spiking networks learn slowly with noisy, cross-trial firing rates
Source: PLoS Comput Biol. 2025 Jul 21;21(7):e1013224. doi: 10.1371/journal.pcbi.1013224 (PMC12367184; doi:10.1371/journal.pcbi.1013224)
Supplement: Attachment — Submitted filename: response.pdf [file pcbi.1013224.s009.pdf]

We thank the reviewers for their comments, which we address below. We additionally made two changes to improve presentation: updated title to better reflect main thrust of paper in light of reviewer feedback, and combined tables 4 and 5 into one table (Table 4). We have included a diff'd PDF with the changes tracked to facilitate an easier review by the referees, and look forward to hearing their reply.

Reviewer #1: I believe that the paper is in an acceptable stage for publication.

I would like to thank the authors for taking my comments into considerations, although my original review was harsh and badly written. I am sorry for that.

We thank the referee for their thorough review. Their comments have made the manuscript much stronger, both initially and in this subsequent revision.

I am still uneasy with the usage of the word "equivalent" in Introduction and Results. Probably for similar reasons as reviewer 2. If used, this term should be given a contextual definition that is mathematically clear and unambiguous, because a naive reader could understand that there exist some kind of general and standard equivalence between LIF and rate. This is not true in general. As explained in Material and Methods, the "equivalence" that is used here comes mathematically from the derivation using stationary inputs. This should be spelled out early in the Results, I am not really satisfied with the current sentences line 93 to 97 which are mathematically vague and imprecise. Hence I suggest the following change:

Suggestion: line 95 one could add a sentence in the spirit of: "[... range of tasks [6, 18].] This equivalence is derived in Material and Methods using the steady-state firing rate of an isolated LIF neuron with constant input current, and remarkably the resulting pair of LIF and firing rate networks share intimate properties when trained with FORCE. [By using exactly parameter matched networks,...].

It is up to the authors to apply this correction, or another one but I believe that some clarification early in results is necessary.

We agree with the referees rewording, and have added a slightly reworded version of their recommended statement shown below (and in the diff'd PDF)

To investigate the differences in spiking and rate based neural networks (Fig 1), we ~~leveraged~~ used the FORCE method to train both spiking neural networks and their ~~firing rate equivalent~~ corresponding parameter-matched instantaneous firing rate networks on a range of tasks [6, 18]. The corresponding rate model is derived in Materials and Methods by analyzing the steady-state firing rate of an isolated LIF neuron under constant input current. Remarkably, when trained with FORCE, the resulting LIF and firing rate networks exhibit closely related properties. By using exactly parameter matched networks, we can compare the weight matrix solutions derived from spiking and rate networks to determine if these

We also agree with their general comments on having a definition of equivalence that is clear and unambiguous. Wherever the term equivalent was used, we replaced this with the "corresponding" or "corresponding parameter matched network". Further, we have added a clarifying sentence on the comparison between the LIF spiking and parameter matched rate network, and have made changes to the manuscript title, based on their previous input.

We thank the referee again for their thorough review of our manuscript, and their many helpful suggestions.

Reviewer #2: I thank the authors for their engagement with the reviews and applaud the beneficial changes made to the paper. It is definitely more clear all around.

I believe this paper now has sufficient clarity for a reader to grasp the main points and is on an interesting and important topic. However, I am still hesitant to support immediate publication because I believe that the investigation is left short of completeness. In the rebuttal to my review, the authors point out that:

"the firing rate approximation (interchanging rates with filtered spikes) is more generally true for slowly varying currents through dynamical systems averaging, or when the network filters (e.g. synaptic filters) are sufficiently long"

and later that

"We leave an analytical derivation of this point, which relates the weight magnitude to the stability of the spiking versus the rate network as a future point of work."

I believe that these two comments (and the origin of my questions which prompted them) point to the only hole left in this work. Specifically, that it appears there are specific regimes of parameter space (not just learning rates) in which the equivalence of the rate networks breaks down and that large learning rates push the networks to these regimes. Expanding on this would allow a much stronger take-away from such a work beyond advising smaller learning rates and would elevate its contribution significantly.

This could be achieved by looking into whether in the breakdown cases, the requirement for equivalence (slowly varying currents) is broken and then attempting to define (analytically or empirically) at what limit of weight-magnitude and/or spiking-rate, this breakdown occurs. This could provide a much deeper insight and takeaway from this work. As a casual (throw-away) example of how such a take-away could look, it could indeed be that weight magnitudes are the determining factor - if so, regularisation of weight magnitudes might ensure network equivalence and/or that spike rate must also be regularized. Any such outcome would turn this work into a seriously depthful contribution.

Given that I don't see that there are any problems with this work, but instead that there is greater potential here, I have opted to submit a recommendation of 'minor revision' so that the editor and authors can consider whether this is sufficiently important for them. I would be okay with an outright acceptance at this stage but, as I mentioned, would be even happier to see this work reach its potential for impact.

We thank the referee for their second review of our manuscript, as both in the first round of revisions, and now, the manuscript has improved substantially thanks to their suggestions. To address their point (and referee 1s), we have replaced the word “equivalent” with “corresponding” as there are indeed regimes where the equivalence can break down. Second, we have added two derivations that highlight why this happens in the fast learning/slow learning regime. This is shown on page 11 of the diff’d PDF. We explain these derivations verbally here for the referees convenience.

First Derivation: When the learning is slow, (regularization is large and weights are small), the regularization parameter scales the principal components of **r**(t), the neural basis generated by FORCE training to reduce the contribution of components with a smaller magnitude. These are the principal components that correspond to the “noise”/variability induced by spiking, and explain less variance. The first few principal components are 1) correlated between rate and spiking neural networks, and 2) are less impacted by the regularization parameter as they have larger norms, and 3) explain more of the variance in the neural basis r(t).

Second Derivation: For large amounts of regularization, where the weights are smaller, the bias variance decomposition implies that smaller decoder (weight) magnitudes leads to smaller contribution of the variance to the overall error.

These two derivations highlight when there’s a general correspondence between the two spiking and rate networks and reveal why there’s a breakdown in this correspondence: when the regularization is small, the smaller magnitude principal components are also used in both the spiking and rate networks. These are uncorrelated, which breaks the correspondence between the learned networks.

We again thank the referee for their review of our work.
